# *In vitro* Effects of Selected Saponins on the Production and Release of Lysozyme Activity of Human Monocytic and Epithelial Cell Lines

**DOI:** 10.3797/scipharm.1012-15

**Published:** 2011-04-18

**Authors:** Racha Helal, Matthias F. Melzig

**Affiliations:** Institute of Pharmacy, Freie Universität Berlin, Königin-Luise-Str. 2+4, 14195, Berlin, Germany

**Keywords:** Lysozyme activity, Saponins, THP-1, HT-29, Fluorescence-based assay

## Abstract

Lysozyme is one of the most important factors of innate immunity and a unique enzybiotic in that it exerts not only antibacterial activity, but also antiviral, anti-inflammatory, anticancer and immunomodulatory activities. The purpose of the present study was to investigate whether *in vitro* exposure to saponins can affect the release and production of lysozyme activity in human monocytic cells THP-1, and in human epithelial cells HT-29. Lysozyme activity levels in cell culture fluids were measured using highly sensitive fluorescence-based lysozyme activity assay. Majority of the examined saponins were demonstrated to stimulate significantly the release of lysozyme activity of monocytes and epithelial cells after one hour treatment at non-toxic concentrations. On the contrary, cells treated with saponins for longer periods up to 72 hours showed tendency to decrease in the secretion and production of lysozyme activity. However, these inhibitory effects of saponins observed with long-term treatment periods were mostly associated with toxic effects of saponins to cells. The results suggested positive contribution of some saponins to lysozyme release of monocytes and epithelial cells upon short exposure. Furthermore, demonstrated ability of these saponins to enhance the release of lysozyme activity can present a new mechanism contribute to explaining important biological characteristics of saponins, including the antibacterial, antiviral, anti-inflammatory or immune-stimulating properties.

## Introduction

Human lysozyme (1,4-*N*-acetylmuramidase, E.C.3.2.1.17) is a ubiquitous low molecular–weight enzyme present in a wide range of biological fluids and tissues within animal and plant kingdoms [1]. Since the initial discovery of lysozyme in 1922 in tears and nasal secretions by Fleming [2], huge literature has accumulated on its structure, function, genetics, biosynthesis, regulation, enzyme activity and properties. Lysozyme is one of the most important factors of innate immunity, possessing antibacterial, antiviral, antitumor and immune modulatory activities [5]. Among major cell types responsible for the production of lysozyme are monocytes/macrophages and neutrophilic granulocytes as a part of their surveillance functions in the immune system [3]. In addition, this antimicrobial peptide tends to be found abundantly in animal tissues that are likely to come in contact with pathogens, such as airway epithelia and other epithelial tissues [4].

The purpose of the present study was to investigate whether in vitro exposure to various concentrations of selected saponins can affect the release and production of lysozyme activity in human monocytic cells THP-1, and in human epithelial cells HT-29. The selection of the investigated saponins was based on their proven antibacterial, antiviral, anti-inflammatory or immune-stimulating properties.

Saponins are steroid or triterpenoid glycosides found in plants, lower marine animals and some bacteria [6, 7]. Saponins have been proved to have immunomodulating effects as well as antitumoral, antiviral and antimicrobial activity [8, 9]. They not only have stimulatory effects on the components of specific immunity, but also present some non-specific immune reactions such as inflammation [10, 11] and monocyte proliferation [12, 13]. Specific saponins have been reported to have the ability to modulate the cell mediated immune system as well as to enhance antibody production and have the advantage that only a low dose is needed for adjuvant activity [14]. The mechanisms of immune-stimulating action of saponins have not been clearly understood, but many explanations have been put forward. Some saponins have reportedly induced the production of cytokines such as interleukins and interferons that might mediate their immunostimulant effects [15, 16].

To investigate the possibility that lysozyme might serve as a useful marker of the immune modulatory properties of selected saponins and to examine the relative contribution of these natural substances to lysozyme production and release in monocytes and epithelial cells, lysozyme activity levels in culture fluids of these two cell types were measured in the present study using highly sensitive fluorescence-based assay [17].

## Results and Discussion

### Human monocytic cells THP-1

#### Secretion of lysozyme activity in cell cultures after short-term treatment with saponins

**Fig. 1.**
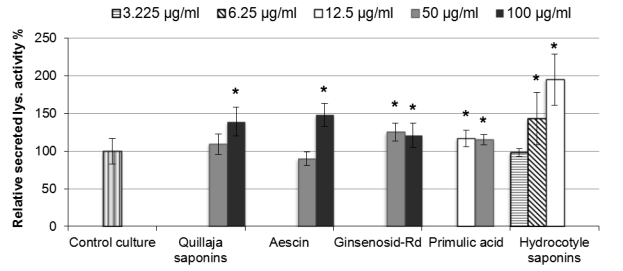
Percent change in the secreted lysozyme activity of 1×10^+6^ cells/ml THP-1 cell cultures treated with various saponins for 1 hour using sensitive fluorescence-based lysozyme activity assay. Control cultures without saponin addition were considered to have 100% lysozyme activity. All points were measured in triplicate, and each column is representative of average value of six separate experiments. Values with asterisk are significantly different (P<0.05, Mann-Whitney U test) from values of control cultures.

Most examined saponins, which have been reportedly known for their influence on the immune response, stimulated the release of lysozyme activity after one hour incubation with monocytes (Fig. 1).

Dose-dependent effect on lysozyme activity secreted in THP-1 cultures was observed with saponins extracted from *Hydrocotyle vulgaris*. 3.125μg/ml final concentration of this saponin had no significant effect, while 6.25μg/ml led to significant increase of 43% in the secreted lysozyme activity and 12.5μg/ml caused further significant increase of 95% after one hour incubation with cells. However, 12.5μg/ml of *Hydrocotyle* saponins started to show slight toxic effects to THP-1 cells that were statistically non-significant after one hour treatment, whereas lower concentrations were non-toxic to cells.

Aescin and saponins from *Quillaja saponaria* bark at 100μg/ml caused statistically significant increases of about 50% and 40%, respectively, in the secreted lysozyme activity of monocytes after one hour treatment compared to control cultures, whereas no significant effects were observed with lower concentrations. None of these two saponins showed significant toxic effects to the cells after one hour exposure at concentrations equal to or lower than 100μg/ml.

Ginsenosid-Rd and primulic acid induced slight but significant increases in the release of lysozyme activity when incubated with cells for one hour.

Ginsenosid-Rd stimulated lysozyme activity release about 25% higher than the control cultures after one hour treatment with no toxic effect, while primulic acid stimulated lysozyme activity release 15% higher than control cultures. No increases in the stimulatory effects of ginsenosid-Rd or primulic acid on lysozyme activity secretion could be detected with the increasing saponin concentrations. Nevertheless, primulic acid at 50μg/ml illustrated slight toxic effects on THP-1 cells that were statistically non-significant after one hour treatment, while lower concentrations were non-toxic to cells.

Another group of saponins showed no influences on lysozyme activity secreted in THP-1 cell cultures after one hour treatment, including hederacosid C, glycyrrhizin, saponins from *Gypsophila paniculata* (saponinum album) and from *Helianthus annuus* marginal flowers. Furthermore, no significant differences were found when these saponins were used at different end-concentrations up to 100μg/ml.

#### Synthesis & secretion of lysozyme activity in cell cultures after long-term treatment with saponins

Lysozyme activity levels in THP-1 cultures supplemented with different saponins were measured at various incubation time points of up to 48 hours.

Contrary to the tendency towards increased lysozyme secretion observed with the short-term treatment with saponins, THP-1 cells treated with saponins for longer incubation periods up to 48 hours showed tendency to decrease in the secretion and production of lysozyme activity in the examined cultures.

Aescin *and Quillaja saponaria* saponins had statistically significant inhibitory effects on the total lysozyme activity synthesized by THP-1 cells as well as on the release of lysozyme in cell cultures treated with each of these two saponins for 24 hours and for 48 hours, whereas *Hydrocotyle vulgaris* and *Gypsophila paniculata* saponins (Saponinum album) had significant inhibitory effects on both total and secreted lysozyme activity only after 48 hours treatment (Tab. 1). On the other hand, primulic acid (6.25μg/ml) and glycyrrhizinic acid (25μg/ml) caused about 18% significant decrease in the total lysozyme activity expressed by THP-1 cells after 24 hours treatment, while this inhibitory activity disappeared after 48 hours treatment.

Another group of saponins showed no influences on total and secreted lysozyme activity of monocytes after long-term treatment, including ginsenosid-Rd, hederacosid C ***and saponins from*** *Helianthus annuus* marginal flowers. Furthermore, no significant differences were found when these saponins were used at different end-concentrations up to 25μg/ml.

### Human colon epithelial cells HT-29

#### Secretion of lysozyme activity in cell cultures after short-term treatment with saponins

**Fig. 2.**
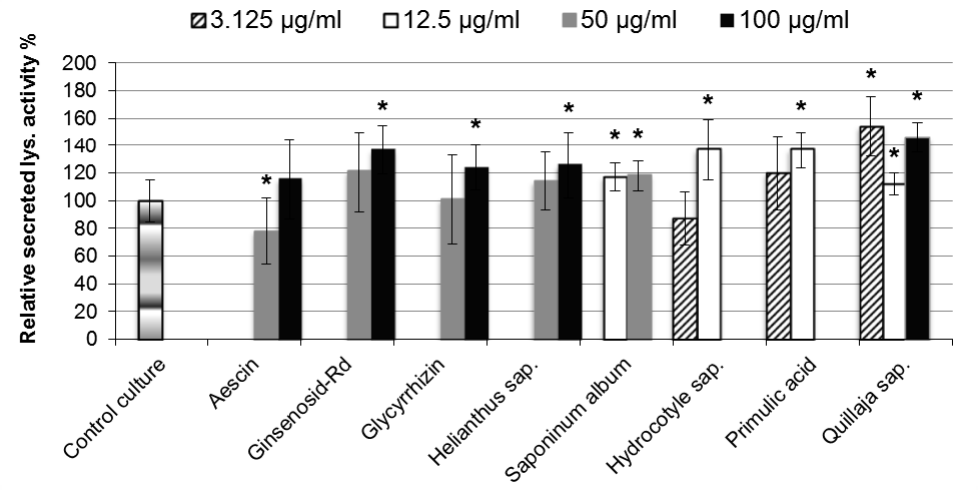
Percent change in the secreted lysozyme activity of 0.5×10^+6^ cells/ml HT-29 cell cultures treated with various saponins for 1 hour using sensitive fluorescence-based lysozyme activity assay. Control cultures without saponin addition were considered to have 100% lysozyme activity. All points were measured in triplicate, and each column is representative of average value of six separate experiments. Values with asterisk are significantly different (P<0.05, Mann-Whitney U test) from values of control cultures.

Majority of the examined saponins stimulated lysozyme secretion after one hour incubation with epithelial cells (Fig. 2).

Saponins of *Quillaja saponaria,* primulic acid and saponinum album stimulated the release of lysozyme activity with no clear dose-effect relationship.

*Quillaja saponaria* saponins at non-toxic concentrations of 3.125μg/ml and 100μg/ml induced significant increases of 54% and 46% in the secreted lysozyme activity respectively, while 12.5μg/ml of this saponin caused less but significant increase of 12%.

Primulic acid caused a significant increase of 26% at a concentration of 25μg/ml, while lower concentration of 12.5μg/ml led to more significant increase of 37%. However, primulic acid at 25μg/ml exhibited slight toxicity to cells that was statistically non-significant after one hour treatment, while lower concentrations showed no cytotoxicity.

Saponins from *Gypsophila paniculata* (Saponinum album) caused slight but significant rise of about 18% in the release of lysozyme activity, with no significant difference when two non-toxic concentrations of 12.5μg/ml and 50μg/ml of the saponin were used.

A direct dose-effect relationship could be found to some extent with saponins from *Hydrocotyle vulgaris*, ginsenosid-Rd, glycyrrhizinic acid and *Helianthus annuus* saponins.

*Hydrocotyle vulgaris* saponins at 12.5μg/ml induced a significant increase of 37% in the secreted lysozyme, while lower doses of 3.125μg/ml and 6.25μg/ml of this saponin had no significant effects. A point to be considered that 12.5μg/ml of *Hydrocotyle* saponins showed slight toxic effects to the epithelial cells that were statistically non-significant, whereas lower concentrations were non-toxic.

Ginsenosid-Rd, glycyrrhizinic and acid *Helianthus annuus* saponins at the non-toxic concentration of 100μg/ml resulted in significant increases of 37%, 24% and 26% in the release of lysozyme activity, respectively. Lower concentrations of 50μg/ml and 25μg/ml had no significant results on lysozyme secretion.

Hederacosid C was the only saponin with no statistically significant effects on the release of lysozyme activity regardless of the saponin concentration used (Data not shown).

The exceptional saponin tended to inhibit lysozyme activity release was aescin. However, this inhibitory effect was particularly significant at the non-toxic concentration of 50μg/ml, whereas higher concentrations of this saponin had no significant effects on lysozyme secretion.

#### Synthesis & secretion of lysozyme activity in cell cultures after long-term treatment with saponins

Lysozyme activity levels in HT-29 cultures supplemented with different saponins were measured at various incubation time points of up to 72 hours.

Contrary to the tendency towards increased lysozyme secretion observed with the short-term treatment with saponins, epithelial cells treated with saponins for 24 hours showed tendency to decrease in the secretion and the expression of lysozyme activity in the examined cultures. However, this inhibitory effect was mostly associated with toxic effects of the saponins to cells.

Primulic acid at concentrations higher than 12.5μg/ml as well as *Hydrocotyle vulgaris* saponins at concentrations equal to or higher than 6.25μg/ml were toxic to the epithelial cells with distinct inhibition of lysozyme activity of cells. Concentration of 3.125μg/ml of these two saponins, which was non-toxic to cells, caused slight decreases in lysozyme activity of cell cultures after 24 hours incubation (Tab. 2).

Furthermore, aescin and saponinum album (from *Gypsophila paniculata*) at 50μg/ml exhibited toxic effects to the cells after 24 hours treatment, which led to significant decreases of total and released lysozyme activity. These two saponins at lower non-toxic concentrations resulted in slight decreases of lysozyme activity (Tab. 2).

*Quillaja saponaria* saponins at non-toxic concentration of 50μg/ml caused significant decreases of 17% and 27% of both total and secreted lysozyme activity respectively after 24 hours incubation with epithelial cells (Tab. 2). Lower concentrations of *Quillaja* saponins had no effects on lysozyme activity.

Another group of the selected saponins showed no significant influences on the production and release of lysozyme activity of the epithelial cells after 24 hours exposure regardless of the used non-toxic doses, including hederacosid C, *Helianthus annuus* saponins, ginsenosid-Rd and glycyrrhizinic acid.

Influence of longer treatment periods up to 72 hours on the expression of active lysozyme of epithelial cells was investigated using glycyrrhizinic acid. As a result, no significant effects of the saponin were found after long exposure to cells up to 72 hours regardless of the used non-toxic doses of the saponin.

## Conclusion

Lysozyme has and still receives attention as a model protein for structural, physicochemical, crystallographic, enzymatic, immunological and evolutionary studies. It is well-known as one of the most important factors of innate immunity, since the principal function attributed to lysozyme in most animals is host defense [19].

There has been interest in lysozyme as a “natural” antibiotic [20]. Lysozyme is unique enzybiotic in that it exerts not only antibacterial activity, but also antiviral, anti-inflammatory, anticancer and immunomodulatory activities [21].

Lysozyme activity levels in the culture fluids of THP-1 monocytes and HT-29 epithelial cells were measured after exposure to selected saponins for various treatment periods using highly sensitive fluorescence-based lysozyme activity assay.

An important point to be considered is that lysozyme secretion and production of human cell cultures could not be easily induced upon treatment with various synthetic, natural or bacterial products as proved by our experiments (Data not shown). In addition, most published reports that concerned lysozyme up- or down-regulation after treatment of the cell cultures with synthetic or natural compounds presented mostly conflicting results. These might be due to the characterization of the used cell lines, or the use of different assay methods that measure lysozyme as a protein, such as ELISA, or as an active enzyme, such as the turbidimetric and the fluorometric assay.

Because the level of active lysozyme may be more relevant for elucidating a defensive role for this protein as an important component of the immune system, a sensitive assay method that can determine the active lysozyme in cell culture supernatants, such as the EnzChek^®^ fluorometric assay used in our present investigation, would provide more meaningful results.

Majority of the saponins examined in the present study were demonstrated to stimulate significantly the release of lysozyme activity of monocytes and epithelial cells after one hour treatment with cells at non-toxic concentrations.

The results suggested positive contribution of some saponins to lysozyme release of monocytes and epithelial cells upon short exposure.

Furthermore, demonstrated ability of saponins to enhance the release of lysozyme activity can present a new mechanism that contributes to explaining important biological characteristics of saponins, including the antibacterial, antiviral, anti-inflammatory or immune-stimulating properties.

The surface-active characteristics of saponins could partly suggest a mechanism of action for the positive contribution of specific saponins to lysozyme secretion of saponin-treated cultures of human monocytes and epithelial cells. Nevertheless, this mechanism cannot adequately explain the stimulatory effects of these saponins, since there are other surface-active saponins examined in this report that have not been able to induce lysozyme secretion of human cells.

On the other hand, the stimulatory effects of these saponins on lysozyme secretion of the examined human cells might be due to one or more components of these saponins that can affect specific cell receptors. Nevertheless, this hypothesis needs further investigations

Further possible mechanisms responsible for the increased levels of secreted lysozyme in some saponin-treated cultures might be through either increased lysozyme synthesis or decreased lysozyme degradation. However, these two mechanisms should be excluded in the short-term treatment experiments, since one hour treatment period has not been sufficient to affect lysozyme transcription, translation or catabolism.

The stimulatory effects of specific saponins on lysozyme release in human cells have to be further investigated through extensive studies on the molecular mechanisms.

Furthermore, the results obtained in the present study can provide the basis for further studies *in vivo* involve investigating lysozyme levels in blood and biological fluids (saliva, gastric juice, duodenal contents) in course of the treatment with herbal drugs that contain these saponins. Elevated lysozyme levels in body fluids can suggest *in vivo* evidence for the immune-stimulatory activity of these natural products among many other biological activities of lysozyme.

The stimulation of immunological system, through elevation of lysozyme activity levels, introduced the specific saponins examined in this report as potential immunostimulant agents from natural origin through lysozyme, the important defense molecule of the innate immune system which is able to control the growth of susceptible bacteria and to modulate host immunity against infections and depressions of immune responses.

## Experimental

### Materials

#### Cell lines & culture medium

Human leukemia cell line THP-1 and human colon epithelial cell line HT-29 were purchased from German collection of microorganisms and cell cultures (DSMZ) Braunschweig, Germany.

Sterile RPMI-1640 medium without phenol red, PBS, L-Glutamine, and fetal bovine serum were obtained from Biochrom, Germany.

Aprotinin from bovine lung, obtained from Sigma, was dissolved in phosphate buffer (pH 7) in a 10 mg/ml (7.3 mM) stock solution, and then diluted with the culture medium to a final concentration of 2 μg/ml (0.3 μM).

#### Kits

EnzChek^®^ Lysozyme Assay Kit was purchased from Molecular Probes™ (Invitrogen Detection Technologies, USA). DQ lysozyme substrate (*Micrococcus lysodeikticus*) stock suspension (1.0mg/ml) and 1000units/ml lysozyme stock solution were prepared according to the manufacturer.

#### Saponins

Ginsenosid-Rd, glycyrrhizinic acid, hederacosid C and saponins of *Quillaja saponaria* bark were purchased from Carl Roth GmbH, Germany. Saponinum album (isolated from *Gypsophila paniculata* L.) was purchased from Merck, Germany. Primulic acid (isolated from *Primula officinalis* L.) was purchased from Fluka AG, Germany. Saponins of *Helianthus annuus* L. were isolated from air-dried marginal flowers by Prof. Zieschang, Humboldt-Universität Berlin, Germany. Aescin was prepared according to Ph. Eur. standards. Saponins from *Hydrocotyle vulgaris* L. were isolated and identified by Prof. Heller, Humboldt-Universität Berlin, Germany.

Each examined saponin was dissolved in PBS in a 1mg/ml stock solution, and then diluted with culture medium to the final concentrations.

### Cell culture

The human leukemia nonadherent cell line THP-1 and human colon epithelial cell line HT-29 were maintained in RPMI-1640 medium without phenol red, supplemented with 10% fetal bovine serum and L-glutamine. Incubation was carried out at 37°C in a humidified atmosphere of 5% CO_2_/95% air. Cell cultures were passaged every 3–4 days. Passages from 5 to 30 were used for the experiments. Cell counts and viability were determined with Casy^®^ cell counter (Schärfe System GmbH).

### Incubation of cell cultures with saponins

THP-1 and HT-29 cells were counted, viability determined and pelleted by centrifugation. Cells were seeded into 24-well plates at an initial concentration of 1.0x10^+6^ cells/ml for monocytic cells and 0.5x10^+6^ cells/ml for epithelial cells. Incubation was carried out at 37°C in a humidified atmosphere of 5% CO_2_ for various incubation periods up to 72 hours in culture medium, or in preparations containing culture medium and one of the examined saponins in question. Cell cultures containing PBS (solvent of the examined saponin) served as the respective controls.

Aprotinin was added to the incubation culture medium of THP-1 at a final concentration of 2μg/ml (0.3μM), in order to achieve best lysozyme determination results, as proved by previous experimental results [18]. With HT-29 cell line, aprotinin addition to culture medium was not necessary, because it had no enhancing effects on the lysozyme activity assay results (Data not shown).

#### Determination of secreted lysozyme activity

Supernatants of the examined and control cell cultures were collected at various time points according to the incubation period examined, and used to determine lysozyme activity released into culture medium.

#### Determination of total lysozyme activity using cell lysis

Cell lysis was needed to facilitate the cell membrane breakage and the release of intracellular lysozyme activity in order to determine total lysozyme activity synthesized by cells. This procedure was performed using two freeze-thaw cycles as following: cell cultures were rapidly frozen using liquid nitrogen, then immediately thawed in 37°C water bath, and vortexed briefly to help lyse cells. This freeze-thaw cycle was repeated two times to completely extract the lysozyme from inside the cells. By lysozyme assay of the whole cell lysates we determined the total amount of lysozyme activity, including lysozyme released in the culture medium and intracellular lysozyme.

### Determination of cell viability

In order to determine the cytotoxicity of saponins, viability of cells after exposure to each saponin for the various incubation periods was measured using CASY^®^ cell analyzer system. Cell viability is assessed based on the integrity of plasma membrane: the living cells have intact plasma membranes whereas membranes of dead cells are broken. When a cell is exposed to a low voltage field, the electric current cannot go though the intact membrane, which is an electric insulator, if it is viable. Otherwise, as the cellular membrane is broken, electric field can go through the injured cell as there are pores on their membrane. Dilution of the cell suspension sample was carried out using freshly filtered isotonic diluting solution for cell cultures specially developed for CASY^®^ analyser (CASYton^®^).

### Fluorescence-based lysozyme activity assay

Lysozyme was quantified according to a sensitive fluorescence-based method using EnzChek^®^ kit described previously [17]. Briefly: Using 96-well black microplate, 50μl volume of cell culture supernatant was used for each reaction. Culture medium without cells was used as a control. Starting the reaction 50μl of the DQ lysozyme substrate working suspension was added to each microplate well containing the experimental or the standard curve samples. Fluorescence intensity of each reaction was measured every 5min to follow the kinetic of the reaction at 37°C for 60min, using fluorescence microplate reader with fluorescein filter (Tecan Austria GmbH). Digestion products from the DQ lysozyme substrate have an absorption maximum at ∼494nm and a fluorescence emission maximum at ∼518nm. Lysozyme activity levels of the experimental samples were determined from the standard curve.

Lysozyme standard curve was linear with a correlation coefficient mean of R^2^ = 0.9962. The rate of lysis of fluorescein labeled *Micrococcus lysodeikticus* suspension induced by lysozyme standard at different concentrations, represented in the slop values of the kinetic curve of each concentration, was linear in the range of 4 – 63 units/ml (Data not shown).

### Statistical analysis

Data in tables and figures were presented as mean ± SD. Differences between groups were assessed by the Mann-Whitney U test. A probability of p < 0.05 was considered significantly different.

## Figures and Tables

**Tab. 1.** Percent change (to control) in the total and secreted lysozyme activity of 1×10^+6^ cells/ml THP-1 cell cultures treated with various saponins for 24 and 48 hours.

**Saponin**	**Conc. (μg/ml)**	**After 24h incubation**			**After 48h incubation**		
		**Secreted lys.^a^** **(%^b^)**	**Total lys.^a^** **(%^b^)**	**Cell viability (%^c^)**	**Secreted lys.^a^** **(%^b^)**	**Total lys.^a^** **(%^b^)**	**Cell viability (%^c^)**
*Quillaja* sap.	6.25	−20 ±7*	−35 ±2*	89 ±8	−21 ±11*	−12 ±4*	88 ±4
Aescin	25	−27 ±6*	−38 ±4*	91 ±5	−16 ±4*	−22 ±5*	90 ±8
*Hydrocotyle vulg.* sap.	3.125	−7 ±16	−13 ±27	90 ±5	−23 ±8*	−25 ±2*	87 ±12
Saponinum album	12.5	−6 ±21	−15 ±22	90 ±6	−21 ±2*	−26 ±3*	89 ±10

a All points were measured in triplicate using sensitive fluorescence-based lysozyme activity assay, and each value is representative of average of at least six separate experiments (±SD);

b Control cultures without saponin addition were considered to have 100% lysozyme activity;

c Cell viability of saponin-treated cultures was calculated by comparison with the control cultures for the same incubation period;

* Values with asterisk are significantly different (P<0.05, Mann-Whitney U test) from values of control cultures.

**Tab. 2.** Percent change (to control) in the total and secreted lysozyme activity of 0.5×10^+6^ cells/ml HT-29 cell cultures treated with various saponins for 24 hours.

**Saponin**	**Conc. (μg/ml)**	**Secreted lys.^a^** **(%^b^)**	**Total lys.^a^** **(%^b^)**	**Cell viability (%^c^)**
Primulic acid	3.125	−7 ±5 %	2 ±3 %	92 ±5 %
	12.5	−16 ±3 %*	0 ±2 %	89 ±10 %
	25	−71 ±2 %*	−67 ±1 %*	45 ±6 %*

*Hydrocotyle vulg*. sap.	3.125	−11 ±3 %*	−5 ±4 %	92 ±4 %
	6.25	−33 ±2 %*	−25 ±3 %*	79 ±6 %*
	12.5	−80 ±3 %*	−79 ±2 %*	43 ±8 %*

Aescin	25	−10 ±6 %*	−4 ±5 %	90 ±4 %
	50	−30 ±1 %*	−21 ±2 %*	80 ±3 %*

Saponinum album	12.5	−7 ±18 %	−8 ±8 %	93 ±7 %
	50	−59 ±1 %*	−53 ±2 %*	47 ±8 %*

*Quillaja* sap.	3.125	−6 ±1 %	1 ±3 %	95 ±3 %
	50	−27 ±1 %*	−17 ±2 %*	90 ±7 %

a All points were measured in triplicate using sensitive fluorescence-based lysozyme activity assay, and each value is representative of average of at least six separate experiments (±SD);

b Control cultures without saponin addition were considered to have 100% lysozyme activity;

c Cell viability of saponin-treated cultures was calculated by comparison with the control cultures for the same incubation period.

* Values with asterisk are significantly different (P<0.05, Mann-Whitney U test) from values of control cultures.

## Abbreviations

(conc.);: Concentration
(HT-29);: human colon adenocarcinoma cell line
(lys.);: lysozyme
(PBS);: phosphate buffered saline
(sap.);: saponin
(SD);: standard deviation
(THP-1): human acute monocytic leukemia cell line.
